# Effect of Vitamin D Supplementation on *In Vitro* Fertilization Outcomes: A Trial Sequential Meta-Analysis of 5 Randomized Controlled Trials

**DOI:** 10.3389/fendo.2022.852428

**Published:** 2022-03-17

**Authors:** Xiaoting Zhou, Xiaomei Wu, Xi Luo, Jingyi Shao, Dongqun Guo, Bo Deng, Ze Wu

**Affiliations:** ^1^ Reproductive Medical Center of Yunnan Province, The Affiliated Hospital of Kunming University of Science and Technology, Kunming, China; ^2^ Department of Reproductive Medicine, The First People’s Hospital of Yunnan Province, Kunming, China; ^3^ Medical School, Kunming University of Science and Technology, Kunming, China; ^4^ Faculty of Life Science and Technology, Kunming University of Science and Technology, Kunming, China; ^5^ Department of Urology, The Second Affiliated Hospital of Fujian Medical University, Quanzhou, China

**Keywords:** *in vitro* fertilization, vitamin D deficiency, vitamin D supplementation, meta-analysis, trial sequential analysis

## Abstract

Despite numerous studies indicating an imperative role of vitamin D for reproduction, the importance of vitamin D supplementation on *in vitro* fertilization (IVF) outcomes remains controversial. We therefore performed this meta-analysis to investigate the IVF outcomes of vitamin D supplementation in infertile women with vitamin D deficiency. We systematically searched PubMed, Embase and the Cochrane library for identifying all relevant studies published before August 2021. Pregnancy rate was defined as the primary outcome while good quality embryo, fertilization rate, ongoing pregnancy, and miscarriage were secondary outcomes. We used Review Manager 5.3 (RevMan) to conduct meta-analysis and examined the robustness of the primary outcome by trial sequential analysis. Five studies were included in the final analysis and it suggested that vitamin D supplementation was associated with improved chemical pregnancy rate (risk ratio [RR] = 1.53, 95% confidence interval [CI] = 1.06 to 2.20, p = 0.02) but not benefited in improving clinical pregnancy rate (RR = 1.34, 95% CI = 0.81 to 2.24, p = 0.25) and all secondary outcomes. Trial sequential analysis suggested further studies are needed to confirm this conclusion. We concluded that vitamin D supplementation should be prescribed to improve chemical pregnancy in infertile women with vitamin D deficiency and more studies are required to further confirm this finding.

## Introduction

It is estimated that infertility was reported in approximately 8 to 12% of reproductive-aged couples around the world (1, 2). As a result, more than 8 million babies have been born from assisted reproductive technologies (ARTs), namely, *in vitro* fertilization (IVF) and intracytoplasmic sperm injection (ICSI) worldwide (3); however the rate of live birth delivery remains suboptimal, reporting a rate of 19 to 22% per initiated cycle (4). More importantly, a population that was confirmed with infertility will suffer a series of negative consequences from multidimensional domains, such as physical, emotional, and psychological aspects (5, 6).

Many studies have been performed to address the role of various micronutrients in fertilization (7, 8), of which vitamin D has an impact on human physiology and pathology under the mediation of vitamin D receptor (VDR) (9). Studies revealed that, following VDR activation may have direct or indirect regulatory functions on the expression of a substantial number of genes (10, 11). A severe problem is that, nowadays, vitamin D deficiency is prevalent worldwide (12–15). Meanwhile, vitamin D deficiency has also been demonstrated to be associated with preeclampsia (16), polycystic ovary syndrome (17), endometriosis (18), and miscarriage (19).

Certainly, several experimental, observational and clinical studies have also investigated the role of vitamin D in reproduction (20, 21). For example, Aleyasin et al. performed animal research and found that vitamin D deficiency was related to the decreased chances of pregnancy, increased risk of pregnancy complications, uterine hypoplasia and infertility (22). In clinical trial, Zhao et al. demonstrated that women with appropriate reserves of vitamin D achieved a higher IVF success rate (23). Meanwhile, several systematic reviews (8, 23–26) of observational studies further suggested that women with deficient or insufficient vitamin D have lower chances of IVF success, however whether vitamin D supplementation can improve IVF outcomes is still controversial. Agreeably, some randomized controlled trials (RCTs) (27–31) have also been performed to demonstrate the role of vitamin D in reproduction. Unfortunately, however, most of the published RCTs (27–30) were interventional studies with small sample size, which were underpowered to draw a definitive conclusion. We therefore performed this meta-analysis to demonstrate the effectiveness of vitamin D supplementation on IVF outcomes through collecting all potentially relevant studies.

## Material and Methods

### Study Registration and Design

It is noted that the protocol of the current meta-analysis was not formally registered in a public platform, however we developed the outline according to the methods recommended by the Cochrane handbook for reviewer (32). Moreover, we reported all pooled results followed the Preferred Reporting Items for Systematic Reviews and Meta-Analyses (PRISMA) checklist (33, 34).

### Information Sources

Two investigators independently searched PubMed, Embase, and the Cochrane library for identifying relevant studies published before August 2021. Multiple terms were used to develop the basic search strategy, namely, vitamin D, vitamin D3, cholecalciferol, calciol, reproductive technique, assisted reproductive technique, *in vitro* fertilization, test-tube baby, and intracytoplasmic sperm injection. All terms were combined by using Boolean logic operators. Details of search strategy of each database can be accessed in **Table S1**. We also checked reference lists of all included studies one-by-one in order to add additional studies. Consensus principle was used to address any contradiction between two investigators.

### Selection Criteria

Two independent investigators selected eligible studies according to inclusion criteria as follows: (a) infertile women were confirmed with vitamin D deficiency, indicating a serum level of less than 30 ng/ml (35); (b) vitamin D supplementation was prescribed in study group, and patients in control group received the same management with patients in study group but no vitamin D supplementation; (c) RCTs reported IVF outcomes, namely, pregnancy rate which included chemical and clinical pregnancy rate, good quality embryo, fertilization rate, ongoing pregnancy and miscarriage; (d) full text of individual study has been released, and (e) studies were published in English language.

Meanwhile, we also developed two exclusion criteria as follows: (a) studies were confirmed with ineligible design, such as traditional literature review, case report, or other non-original studies and (b) repeated reports from the same research group and contained insufficient data.

### Study Selection

Two independent investigators conducted the process of selecting eligible studies. Investigators firstly imported citations identified from 3 targets into EndNote v.X9. Then we removed duplicates based on automatic repeated literature detection function. Moreover, we also removed remained duplicate studies which were not detected by literature management software through the manual check. For remaining unique studies, two investigators independently checked its eligibility through screening titles and abstracts. Finally, we accessed the full-texts of all studies for eligibility evaluation. We clearly recorded the number of ineligible studies excluded and corresponding reasons of excluding each study. Consensus principle was used to address any contradiction between two investigators.

### Data Extraction

Two independent investigators extracted the following data, namely, the name, publication year, region, sample size, average age, details of vitamin D supplementation, duration of intervention, outcomes, and information of the first author for assessing the risk of bias. Contact with the corresponding author has not occurred because all essential information was obtained from original studies. Consensus principle was used to address any contradiction between two investigators.

### Outcomes of Interest

In this meta-analysis, we defined pregnancy rate which included chemical and clinical pregnancies as the primary outcome. Of which, chemical pregnancy was defined as serum level of human chorionic gonadotropin beta subunit (β-hCG) >50 IU/L at 14 days after embryo transfer (28), and clinical pregnancy was defined as the presence of at least one intrauterine gestational sac with viable fetus (36). Among 4 secondary outcomes, good quality embryos were defined as the presence of 6 to 8 blastomeres with even size and <25% fragmentation (27), fertilization rate was defined as the result of the number of 2PN observed divided by the number of injected oocytes (27), ongoing pregnancy was defined as pregnancy with detectable heart rate at more than 12 weeks gestation (37), and miscarriage was defined as a positive pregnancy test but no detectable heart rate before 24 weeks gestation (37).

### Risk of Bias Assessment

Two independent investigators appraised the methodological quality of all included studies by using the Cochrane Collaboration’s risks of bias tool (38). In this assessment tool, the methodological quality was assessed from seven items, namely, random sequence generation, allocation concealment, blinding of personnel and participants, blinding of outcome assessment, incomplete outcome data, selective reporting, and other biases. Consensus principle was used to address any contradiction between two investigators.

### Statistical Analysis

We used Review Manager 5.3 (RevMan, version 5.3.5; Nordic Cochrane Centre, The Cochrane Collaboration, Copenhagen, Denmark) to perform all statistical analyses. All outcomes in this study were categorical variables, and thus we used risk ratios (RR) with corresponding 95% CI to estimate the pooled results. Heterogeneity across studies was simultaneously evaluated by using chi square and heterogeneity index (I2). We estimated all pooled results based on a random-effects model considering the fact that it is impossible to completely eliminate the variations across studies. Publication bias was not performed in this meta-analysis because of the accumulated number of eligible studies for individual outcome were not more than ten (38). For all tests, statistical significance was defined as a p <0.05.

### Trial Sequential Analysis

Meta-analysis is a method to estimate the overall effect size through accumulating sparse studies (39). Therefore, the robustness of pooled results in meta-analysis will be significantly impaired as the number of repeatedly accumulating evidence increase. Inspired by sequential analysis in clinical trial, trial sequential meta-analysis was proposed to determine whether a definitive conclusion could be drew based on the available evidence (40). In trial sequential analysis, a required information size (RIS) will be estimated based on available evidence, and meanwhile adjusted monitoring boundary will be generated after adjusting conventional significance level to decrease the risk of false positive results (41). For individual outcome, a definitive conclusion will be obtained if accumulated sample size was more than RIS, or accumulative Z-curve cross through trial sequential monitoring boundary or cross through conventional monitoring boundary and entering into infertility area. In this case, no further study will be required (40). In the present trial sequential meta-analysis, we estimated the diversity-adjusted information size and built O’Brien–Fleming α-spending boundaries by using 5% type I error and 20% type 2 error rate (42), which were two-side values. The heterogeneity correction was automatically entered by software. TSA software version 0.9.5.10 beta was used to perform all trial sequential analyses.

## Results

### Search of Literature

**Figure 1:**
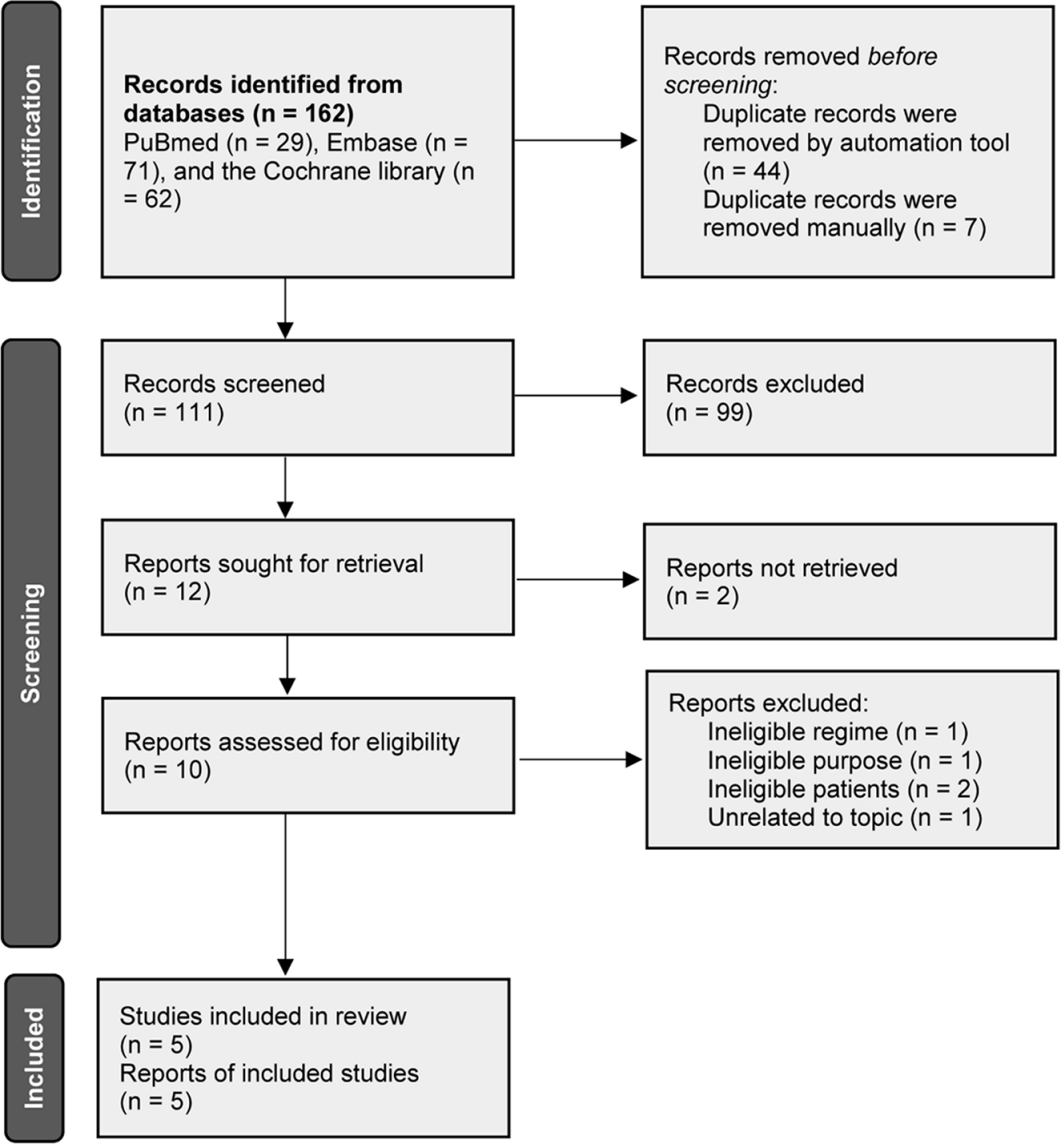
Flow diagram of searching and selecting eligible study in this meta-analysis.

A total of 162 records were initially identified after electronically searching PubMed, Embase and the Cochrane library. The publication time was limited from their inception to August 2021. A total of 111 unique records were persisted to be initially checked for eligibility after excluding 51 duplicate records. We excluded 99 irrelevant records after screening the title and abstract. Among the remaining 12 potentially eligible records, we retrieved full-texts of 10 records because of 2 records were determined to be the conference abstract which reported insufficient information. Finally, 5 studies (27–31) were judged to meet our selection criteria after excluding 5 ineligible studies due to the following reasons, namely, ineligible regime (n = 1), ineligible study aim (n = 1), ineligible patients (n = 2), and unrelated to our topic (n = 1). We used **Figure 1** to display the flow chart of study retrieval and selection.

### Essential Characteristics of All Eligible Studies

Among enrolled 5 eligible studies, 3 studies (27, 28, 30) were performed in Iran and another 2 studies (29, 31) were performed in Italy. The publication time of individual study ranged from 2014 to 2021. Most eligible studies (27–30) enrolled inadequate sample size with a median number of 92, apart from 1 study performed by Somigliana et al. (31), in which 573 patients were included in the final analysis. Two studies (27, 28) instructed patients to take orally vitamin D pearl capsule, 2 studies (29, 31) instructed patients to take orally vitamin D3, and 1 study (30) instructed patients to take orally calcitriol pills. Among included studies, 4 studies (27–30) reported details of treatment duration except for 1 study (31). We used **Table 1** to summarize the essential characteristics of all eligible studies.

**Table 1:** Basic characteristics of 5 eligible studies.

Study	Country	Sample size	Age, yrs	BMI, kg/m^2^	Pretreatment serum vitamin D, (ng/ml)	Details of regimes
Aflatoonian et al. (28)	Iran	51 vs 55	(28.45 ± 3.74) vs (29.56 ± 4.68)	(26.87 ± 1.77) vs (26.29 ± 1.67)	(15.81 ± 5.94) vs (14.2 ± 6.33)	50,000 IU vitamin D pearl capsule weekly, for 6-8 weeks
Abedi et al. (27)	Iran	42 vs 43	(31.9 ± 4.2) vs (30.8 ± 4.4)	(23.9 ± 2.1) vs (23.8 ± 1.9)	n.r.	50,000 IU vitamin D pearl capsule weekly, for 6 weeks
Bezerra Espinola et al. (29)	Italy	50 vs 50	(34.7 ± 6.7) vs (35.9 ± 3.7)	(21.9 ± 2.1) vs (22.0 ± 2.3)	(23.4 ± 6.7) vs (23.4 ± 6.6)	2,000 IU vitamin D3 for 12 weeks
Doryanizadeh, et al. (30)	Iran	36 vs 38	(32.5 ± 4.9) vs (31.6 ± 4.9)	(25.3 ± 3.2) vs (24.9 ± 3.4)	(27.5 ± 1.8) vs (27.6 ± 1.8)	0.25 µg calcitriol pills daily for 4 weeks
Somigliana et al. (31)	Italy	285 vs 288	(35.0 [32.0–37.0]) vs (35.0 [33.0–37.0])	(20.8 [19.5–22.5]) vs (21.1 [19.7–22.9])	(20.0 [15.5–23.6]) vs (19.9 [14.6–23.9])	single dose of 600,000 IU of vitamin D3

yrs, years; CMP, chemical pregnancy; CNP, clinical pregnancy; FR, fertilization rate; GQE, good quality embryo; n.r., not reported.

### Risk of Bias Assessment

**Figure 2:**
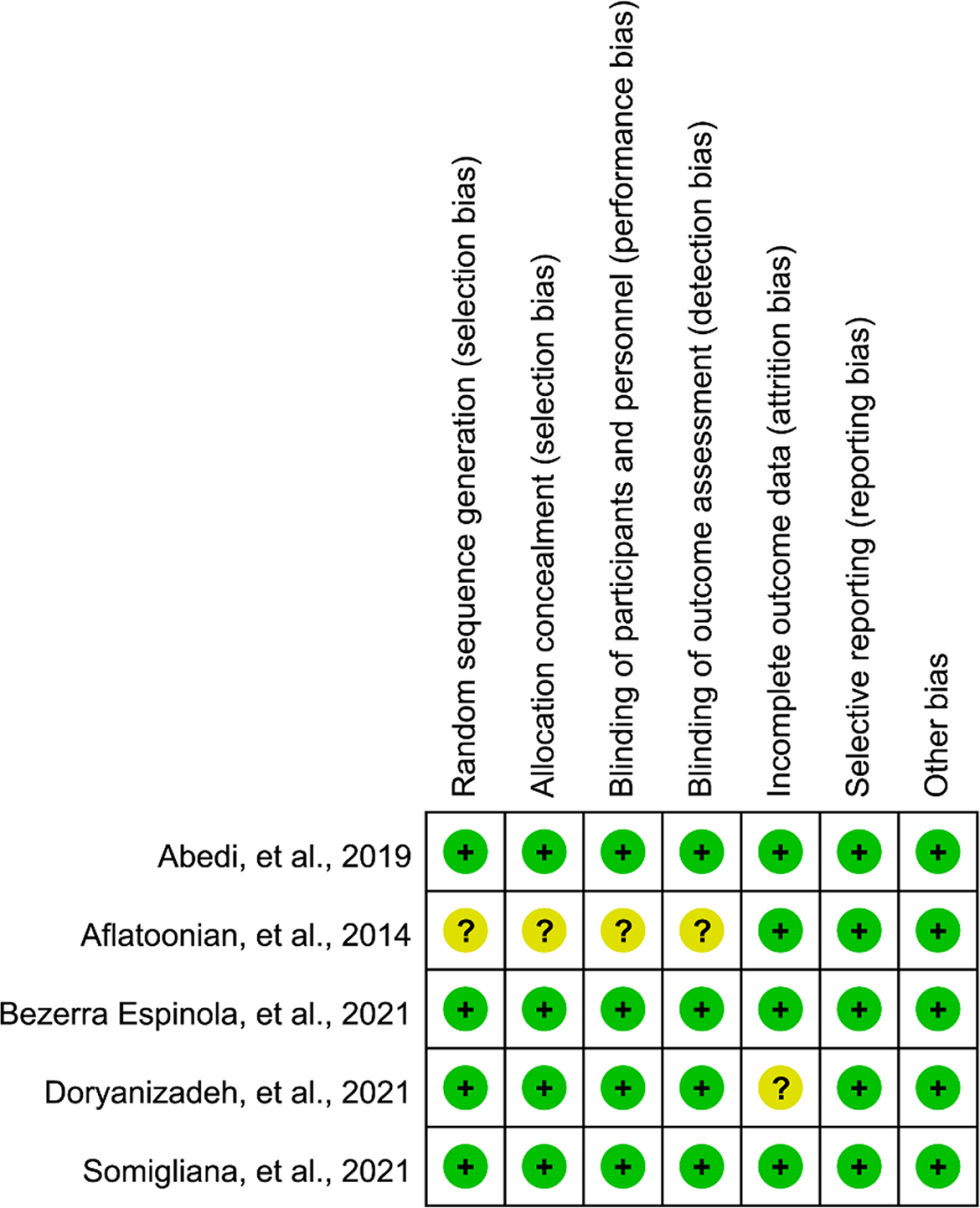
Risk of bias summary of eligible studies.

Details of risk of bias assessment were summarized in **Figure 2**. Among 5 eligible studies, 4 studies (27, 29–31) correctly generated random sequence, concealed allocation, and blinded personnel, patients and outcome assessors, however 1 study (28) did not report details which can be used to accurately judge whether selection bias, performance bias and detection bias were adequately performed. Only 1 study (30) did not provide sufficient information to assess attrition bias, and all studies (27–31) were judged as low risk in remaining two domains, namely, reporting bias and other bias.

### Meta-analysis of Pregnancy Rate

**Figure 3:**
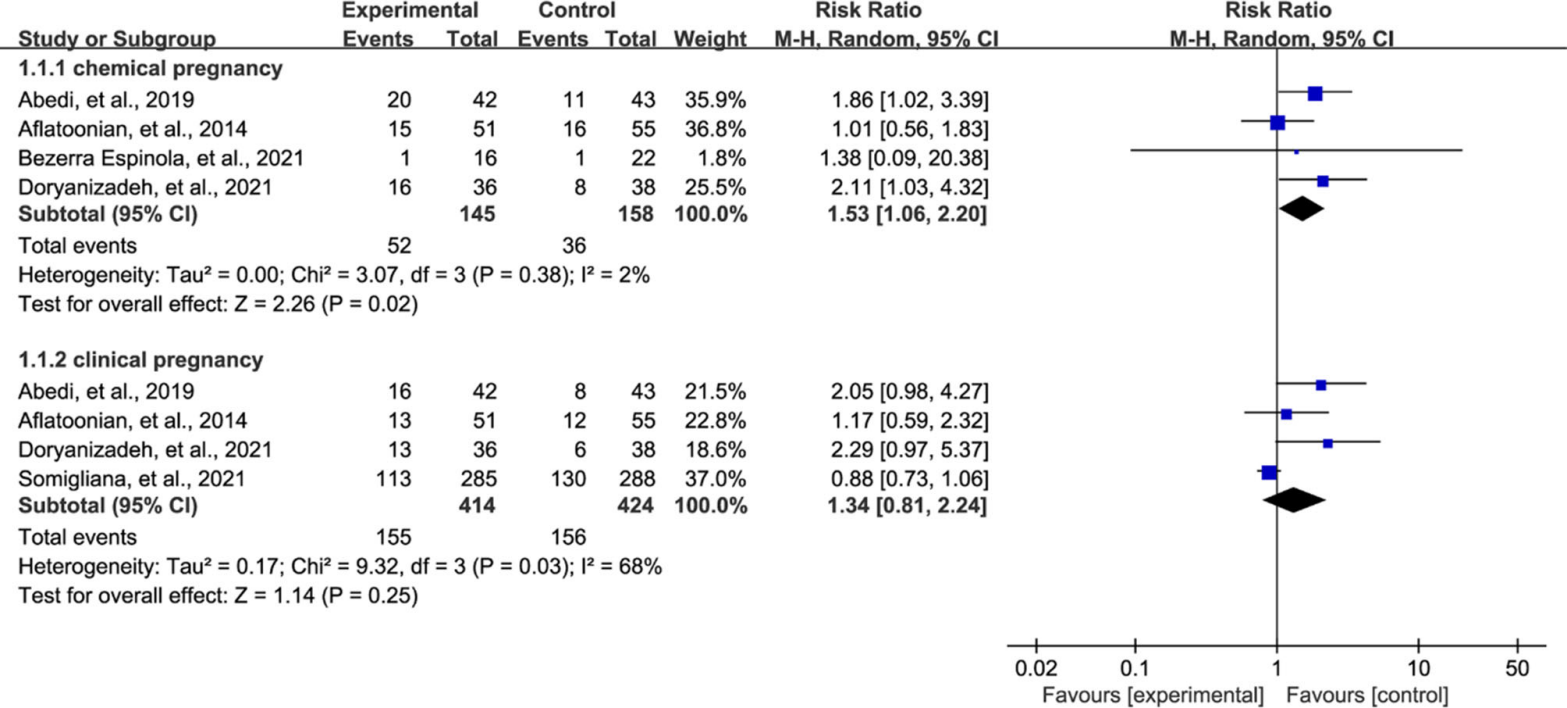
Meta-analysis of pregnancy rate between vitamin D supplementation and no supplementation.

All eligible studies reported pregnancy rate after treatment, however 4 studies (27–30) reported chemical pregnancy and 4 studies (27, 28, 30, 31) reported clinical pregnancy. We therefore separately estimated the pooled results of two outcomes according to the principle of performing subgroup analysis. Meta-analysis suggested that vitamin D supplementation treatment significantly increased the chemical pregnancy rate compared with patients who received control regime (RR = 1.53, 95% CI = 1.06 to 2.20, P = 0.02, **Figure 3**). However, meta-analysis did not detect a significant difference in clinical pregnancy rate between both groups (RR = 1.34, 95% CI = 0.81 to 2.24, P = 0.25, **Figure 3**).

**Figure 4:**
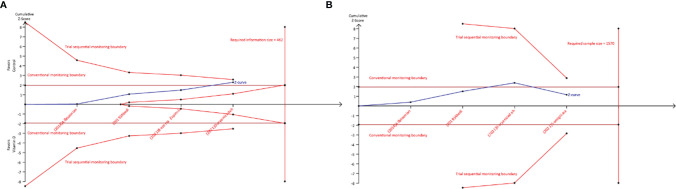
Trial sequential analysis of chemical and clinical pregnancy. **(A)** Required sample size of 462; **(B)** required sample size of 1570.

In trial sequential analysis of chemical pregnancy, we estimated a required sample size (also termed as information size) of 462, and the accumulated sample size in our study did not exceed the required sample size. Moreover, although accumulative Z-curve across through conventional monitoring boundary for benefit after added the fourth study, it did not cross through trial sequential monitoring boundary for benefit (**Figure 4A**). Therefore, we cannot draw a definitive conclusion about whether vitamin D supplementation can benefit to increase chemical pregnancy due to the presence of false positive. For clinical pregnancy, trial sequential analysis estimated a required sample size of 1,570, which greatly more than the accumulated sample size in our study. It is noted that, however, the accumulative Z-curve crosses through conventional monitoring boundary to enter the infertile area (**Figure 4B**) after adding the fourth study, which confirmed the result from meta-analysis.

### Meta-Analysis of Secondary Outcomes

**Figure 5:**
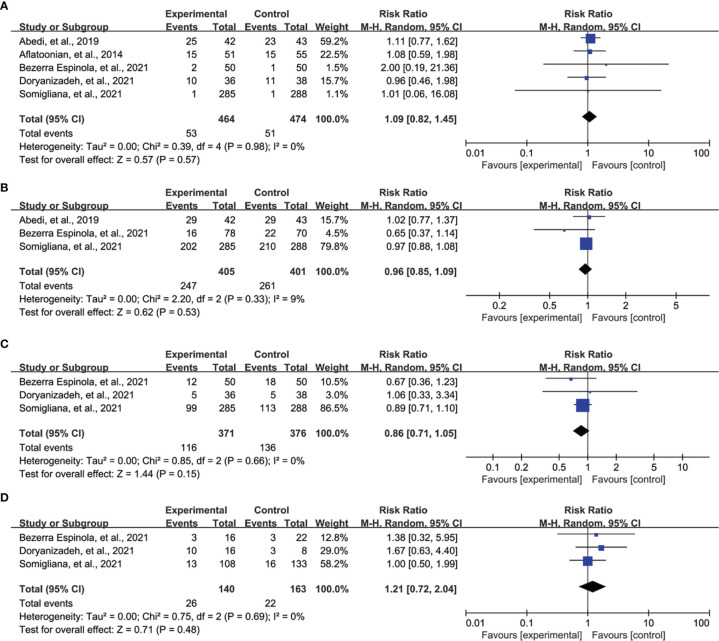
Meta-analysis of secondary outcomes, namely, good quality embryo **(A)**, fertilization rate **(B)**, ongoing pregnancy **(C)**, and miscarriage **(D)**.

All eligible studies reported the data of good quality embryo, and meta-analysis suggested no statistical difference between both groups (RR = 1.09, 95% CI = 0.82 to 1.45, P = 0.57, **Figure 5A**). Three studies reported fertilization rate, and meta-analysis revealed that there was no statistical difference between vitamin D supplementation and control groups (RR = 0.96, 95% CI = 0.85 to 1.09, P = 0.53, **Figure 5B**). Three studies reported ongoing pregnancy, and meta-analysis indicated no statistical difference between both groups (RR = 0.86, 95% CI = 0.71 to 1.05, P = 0.15, **Figure 5C**). Moreover, three studies also reported miscarriage rate, and meta-analysis did not detect a statistical difference between both groups (RR = 1.21, 95% CI = 0.72 to 2.04, P = 0.48, **Figure 5D**).

## Discussion

Infertility is a multifactorial condition, which is affected by several factors, namely, lifestyle, eating habits or nutrition (27). It is noted that the imperative role of vitamin D in fertilization has been investigated (43), and it has been demonstrated to have a critical impact on physiology and pathology (27, 30). It is noted that, actually, the functions of vitamin D were dedicated to the vitamin D receptors (VDRs) (10, 11), which has also been supported by the following three aspects (7): (a) the presence of VDRs in the hypothalamic-ovarian-uterine-placental axis (also named as the reproductive axis), (b) the existence of enzymes involved in hydroxylation, and (c) identification of local synthesis of vitamin D in human placenta and decidua. As a member of the nuclear receptor family of transcription factors, VDRs form a heterodimer with a retinoid-X receptor and bind to hormone response elements on DNA to regulate expression of specific gene products (27). Therefore, the reproductive axis is regarded as one of the target organs for Vitamin D (44). Because of this, vitamin D has been advocated to play a critical role in the biosynthesis of sex hormones and also post fertilization in the process (45) and production of hCG (46).

Currently, several observational studies and subsequent systematic review have correlated low serum vitamin D levels to a reduction of both natural fertility and IVF success (23, 24, 43, 47). Recently, several RCTs have also been performed to further test the effectiveness of vitamin D supplementation on IVF outcomes. Nevertheless, these studies were insufficiently powered to draw definite conclusions. We therefore conducted this trial sequential meta-analysis to firstly evaluated the effectiveness of vitamin D supplementation on IVF outcomes in infertile women through accumulating results from eligible RCTs. Based on the results from the present trial sequential meta-analysis, we found that vitamin D supplementation may have potential ability of improving chemical pregnancy, however this conclusion must be further confirmed because of trial sequential analysis detected the presence of false positive result. It is noted that we found that vitamin D supplementation did not have an additional benefit for the improvement of clinical pregnancy and this finding has also been demonstrated by trial sequential analysis. For good quality embryo, fertilization rate, ongoing pregnancy, and miscarriage, we did not also find statistical benefits in patients received vitamin D supplementation.

Among 4 eligible studies reported clinical pregnancy, 2 studies (27, 30) reported an improved clinical pregnancy, which was consistent with our finding. However, another 2 studies (28, 29) reported inconsistent results that vitamin D supplementation could not improve this outcome. Evidence suggested that high dose of vitamin D3 is expected to properly maintain peripheral levels of vitamin D above 30 ng/ml for 3 months (48), that is, a period that in most cases properly covers a complete IVF cycle. However, the study by Bezerra Espinola et al. instructed patients to take orally 2,000 IU vitamin D3 (29), which may be the reason of leading to inconsistent result. Although another two studies performed by Aflatoonian (28) and Abedi (27) respectively prescribed a dose of 50,000 IU, Abedi instructed patients to oral vitamin D at 6 weeks before intracytoplasmic sperm injection (ICSI) (27), but Aflatoonian prescribed vitamin D after IVF/ICSI with cryopreservation of embryos (28). The difference in the time of supplying vitamin D may be the contributor to the conflicting results. Moreover, study by Abedi et al. used the ICSI method whereas Aflatoonian et al. performed the IVF/ICSI method, which also contributed to the contradiction between two results. Another study by Doryanizadeh et al. reported a larger magnitude for improvement of clinical pregnancy (30) because of calcitriol, which is the most active form of vitamin D and has similar functions to a steroid hormone (25, 43), has been prescribed for patients. Based on this information, calcitriol may be preferentially selected for infertile patients with vitamin D deficiency because of calcitriol can facilitate calcium transfer in the placenta, stimulate lactogen expression, facilitate the decidualization of the endometrium, and regulate HOXA10 gene expression in the process of fertilization (49). However, we must point out that all included studies were underpowered to generate a definitive conclusion, which also be confirmed by our trial sequential analysis. Therefore, this conclusion should be cautiously interpreted.

Meanwhile, a total of 4 eligible studies (27, 28, 30, 31) reported clinical pregnancy, and all studies indicated no statistical difference between both groups, which consistently supported our finding. It is noted that these 4 studies prescribed a high dose of vitamin D (27, 28, 31) or the most active form of vitamin D (30), which enhanced our confidence for findings. Although there were 3 studies enrolled inadequate sample size, a study with a large sample size also generated consistent result (31), and pooled result was also be confirmed by trial sequential analysis. We therefore convinced that vitamin D supplementation may not benefit to clinical pregnancy among infertile women with vitamin D deficiency.

For remaining secondary outcomes, our meta-analysis did not reveal statistical benefit for patients received vitamin D supplementation, which were consistent with those results in enrolled individual study. It is noted that our findings about secondary outcomes were also consistently supported by other studies (50, 51). Moreover, recent investigations by Rudick et al. (7) and Banker et al. (52) on the effect of vitamin D on the cycles of recipients of donated eggs also showed that the eggs were not affected by vitamin D levels in the blood of recipients; therefore, there was no relationship between vitamin D levels and the factors of ovarian stimulation and quality of the embryo. However, these findings should be further tested because of inadequate sample size was accumulated.

Several limitations existed in this meta-analysis must be further interpreted. First and foremost, details of vitamin D varied from one study to another. Specifically, vitamin D pearl capsule was used in 2 studies (27, 28), vitamin D3 was used in 2 studies (29, 31), and calcitriol pill was used in 1 study (30). We did not perform subgroup analysis to investigate the separate effectiveness of individual content on IVF outcomes due to limited number of eligible studies. This difference may impair the robustness of our findings. Second, duration of administration of vitamin D was also different across studies, which may also decrease the reliability of our findings because of subgroup analysis was not performed. Third, all studies were performed in Iran or Italy, and thus it is difficult to expand our findings to other cultural settings.

## Conclusion

Vitamin D supplementation, by improving chemical pregnancy, may significantly increase the chances of successful IVF cycle in infertile women with vitamin D deficiency. However, we also suggest more studies to confirm this finding due to the presence of false positive result. Meanwhile, whether vitamin D administration has positive effects on other IVF outcomes such as fertilization rate and ongoing pregnancy should also be further investigated due to inadequate number of eligible studies. Furthermore, it is imperative to determine which forms of Vitamin D may be optimal during IVF cycle because no study has been performed to investigate the difference between various forms of Vitamin D.

## Data Availability Statement

The original contributions presented in the study are included in the article/**Supplementary Material**. Further inquiries can be directed to the corresponding authors.

## Author Contributions

Conceptualization: ZW. Methodology: XZ and XW. Software: DG. Validation: XL and JS. Formal analysis: XZ and XW. Investigation: XZ and XL. Resources: ZW and BD. Data curation: JS and XL. Writing original draft preparation: XZ. Writing review and editing: XZ. Visualization: XZ. Supervision: BD. Project administration: ZW. Funding acquisition: ZW. All authors listed have made a substantial, direct, and intellectual contribution to the work and approved it for publication.

## Funding

This work was supported by Yunnan Provincial Reproductive and Gynecology Clinical Medicine Center (Grant No. zx2019-01-01).

## Conflict of Interest

The authors declare that the research was conducted in the absence of any commercial or financial relationships that could be construed as a potential conflict of interest.

## Publisher’s Note

All claims expressed in this article are solely those of the authors and do not necessarily represent those of their affiliated organizations, or those of the publisher, the editors and the reviewers. Any product that may be evaluated in this article, or claim that may be made by its manufacturer, is not guaranteed or endorsed by the publisher.
