# Supplementary material for: Effect of Vitamin D Supplementation on In Vitro Fertilization Outcomes: A Trial Sequential Meta-Analysis of 5 Randomized Controlled Trials
Source: Front Endocrinol (Lausanne). 2022 Mar 17;13:852428. doi: 10.3389/fendo.2022.852428 (PMC8969598; doi:10.3389/fendo.2022.852428)
Supplement: Supplementary file 2 [file Table_2.docx]

**Search strategy of PubMed**

| Search number | Query |
| --- | --- |
| 15 | (((("Vitamin D"[Mesh]) OR "Cholecalciferol"[Mesh]) OR ((((((Vitamin D[Title/Abstract]) OR (Cholecalciferol[Title/Abstract])) OR (Cholecalciferols[Title/Abstract])) OR (Calciol[Title/Abstract])) OR (Vitamin D3[Title/Abstract])) OR (Vitamin D 3[Title/Abstract]))) AND ((((("Reproductive Techniques"[Mesh]) OR "Reproductive Techniques, Assisted"[Mesh]) OR "Fertilization in Vitro"[Mesh]) OR "Sperm Injections, Intracytoplasmic"[Mesh]) OR ((((((((((((((((((((((((((Reproductive Technique[Title/Abstract]) OR (Reproductive Techniques[Title/Abstract])) OR (Reproduction Technique[Title/Abstract])) OR (Reproduction Techniques[Title/Abstract])) OR (Reproduction Technic[Title/Abstract])) OR (Reproduction Technics[Title/Abstract])) OR (Reproductive Technology[Title/Abstract])) OR (Reproductive Technologies[Title/Abstract])) OR (Assisted Reproductive Technique[Title/Abstract])) OR (Assisted Reproductive Technic[Title/Abstract])) OR (Assisted Reproductive Technics[Title/Abstract])) OR (Assisted Reproductive Techniques[Title/Abstract])) OR (Assisted Reproductive Technology[Title/Abstract])) OR (Assisted Reproductive Technologies[Title/Abstract])) OR (In Vitro Fertilization[Title/Abstract])) OR (In Vitro Fertilizations[Title/Abstract])) OR (Test-Tube Fertilization[Title/Abstract])) OR (Test Tube Fertilization[Title/Abstract])) OR (Test Tube Fertilizations[Title/Abstract])) OR (Fertilizations in Vitro[Title/Abstract])) OR (Test-Tube Babies[Title/Abstract])) OR (Test-Tube Baby[Title/Abstract])) OR (Test-Tube Babies[Title/Abstract])) OR (Intracytoplasmic Sperm Injection[Title/Abstract])) OR (Intracytoplasmic Sperm Injections[Title/Abstract])) OR (ICSI[Title/Abstract])))) AND ((("Random Allocation"[Mesh] OR "Randomized Controlled Trial" [Publication Type] OR "Randomized Controlled Trials as Topic"[Mesh]) OR "Placebos"[Mesh]) OR (((((random allocation[Title/Abstract]) OR (Randomization[Title/Abstract])) OR (Sham Treatment[Title/Abstract])) OR (placebo[Title/Abstract])) OR (random*[Title/Abstract]))) |
| 11 | (("Random Allocation"[Mesh] OR "Randomized Controlled Trial" [Publication Type] OR "Randomized Controlled Trials as Topic"[Mesh]) OR "Placebos"[Mesh]) OR (((((random allocation[Title/Abstract]) OR (Randomization[Title/Abstract])) OR (Sham Treatment[Title/Abstract])) OR (placebo[Title/Abstract])) OR (random*[Title/Abstract])) |
| 10 | ((((random allocation[Title/Abstract]) OR (Randomization[Title/Abstract])) OR (Sham Treatment[Title/Abstract])) OR (placebo[Title/Abstract])) OR (random*[Title/Abstract]) |
| 9 | ("Random Allocation"[Mesh] OR "Randomized Controlled Trial" [Publication Type] OR "Randomized Controlled Trials as Topic"[Mesh]) OR "Placebos"[Mesh] |
| 6 | (((("Reproductive Techniques"[Mesh]) OR "Reproductive Techniques, Assisted"[Mesh]) OR "Fertilization in Vitro"[Mesh]) OR "Sperm Injections, Intracytoplasmic"[Mesh]) OR ((((((((((((((((((((((((((Reproductive Technique[Title/Abstract]) OR (Reproductive Techniques[Title/Abstract])) OR (Reproduction Technique[Title/Abstract])) OR (Reproduction Techniques[Title/Abstract])) OR (Reproduction Technic[Title/Abstract])) OR (Reproduction Technics[Title/Abstract])) OR (Reproductive Technology[Title/Abstract])) OR (Reproductive Technologies[Title/Abstract])) OR (Assisted Reproductive Technique[Title/Abstract])) OR (Assisted Reproductive Technic[Title/Abstract])) OR (Assisted Reproductive Technics[Title/Abstract])) OR (Assisted Reproductive Techniques[Title/Abstract])) OR (Assisted Reproductive Technology[Title/Abstract])) OR (Assisted Reproductive Technologies[Title/Abstract])) OR (In Vitro Fertilization[Title/Abstract])) OR (In Vitro Fertilizations[Title/Abstract])) OR (Test-Tube Fertilization[Title/Abstract])) OR (Test Tube Fertilization[Title/Abstract])) OR (Test Tube Fertilizations[Title/Abstract])) OR (Fertilizations in Vitro[Title/Abstract])) OR (Test-Tube Babies[Title/Abstract])) OR (Test-Tube Baby[Title/Abstract])) OR (Test-Tube Babies[Title/Abstract])) OR (Intracytoplasmic Sperm Injection[Title/Abstract])) OR (Intracytoplasmic Sperm Injections[Title/Abstract])) OR (ICSI[Title/Abstract])) |
| 5 | (((((((((((((((((((((((((Reproductive Technique[Title/Abstract]) OR (Reproductive Techniques[Title/Abstract])) OR (Reproduction Technique[Title/Abstract])) OR (Reproduction Techniques[Title/Abstract])) OR (Reproduction Technic[Title/Abstract])) OR (Reproduction Technics[Title/Abstract])) OR (Reproductive Technology[Title/Abstract])) OR (Reproductive Technologies[Title/Abstract])) OR (Assisted Reproductive Technique[Title/Abstract])) OR (Assisted Reproductive Technic[Title/Abstract])) OR (Assisted Reproductive Technics[Title/Abstract])) OR (Assisted Reproductive Techniques[Title/Abstract])) OR (Assisted Reproductive Technology[Title/Abstract])) OR (Assisted Reproductive Technologies[Title/Abstract])) OR (In Vitro Fertilization[Title/Abstract])) OR (In Vitro Fertilizations[Title/Abstract])) OR (Test-Tube Fertilization[Title/Abstract])) OR (Test Tube Fertilization[Title/Abstract])) OR (Test Tube Fertilizations[Title/Abstract])) OR (Fertilizations in Vitro[Title/Abstract])) OR (Test-Tube Babies[Title/Abstract])) OR (Test-Tube Baby[Title/Abstract])) OR (Test-Tube Babies[Title/Abstract])) OR (Intracytoplasmic Sperm Injection[Title/Abstract])) OR (Intracytoplasmic Sperm Injections[Title/Abstract])) OR (ICSI[Title/Abstract]) |
| 4 | ((("Reproductive Techniques"[Mesh]) OR "Reproductive Techniques, Assisted"[Mesh]) OR "Fertilization in Vitro"[Mesh]) OR "Sperm Injections, Intracytoplasmic"[Mesh] |
| 3 | (("Vitamin D"[Mesh]) OR "Cholecalciferol"[Mesh]) OR ((((((Vitamin D[Title/Abstract]) OR (Cholecalciferol[Title/Abstract])) OR (Cholecalciferols[Title/Abstract])) OR (Calciol[Title/Abstract])) OR (Vitamin D3[Title/Abstract])) OR (Vitamin D 3[Title/Abstract])) |
| 2 | (((((Vitamin D[Title/Abstract]) OR (Cholecalciferol[Title/Abstract])) OR (Cholecalciferols[Title/Abstract])) OR (Calciol[Title/Abstract])) OR (Vitamin D3[Title/Abstract])) OR (Vitamin D 3[Title/Abstract]) |
| 1 | ("Vitamin D"[Mesh]) OR "Cholecalciferol"[Mesh] |

**Search strategy of Embase**

1 (Vitamin D or Cholecalciferol or Cholecalciferols or Calciol or Vitamin D3 or Vitamin D 3).af.

2 exp vitamin D/

3 exp colecalciferol/

4 2 or 3

5 1 or 4

6 (Reproductive Technique* or Reproduction Technique* or Reproduction Technic* or Reproductive Technology or Reproductive Technologies or Assisted Reproductive Technique* or Assisted Reproductive Technic* or Assisted Reproductive Technology or Assisted Reproductive Technologies or In Vitro Fertilization* or Test-Tube Fertilization* or Test Tube Fertilization* or Fertilizations in Vitro or Test-Tube Babies or Test-Tube Baby or Test-Tube Babies or Intracytoplasmic Sperm Injection* or ICSI).af.

7 exp reproductive procedure/

8 exp infertility therapy/

9 exp in vitro fertilization/

10 exp intracytoplasmic sperm injection/

11 7 or 8 or 9 or 10

12 6 or 11

13 (random allocation or randomization or shame treatment or placebo or random*).af.

14 exp randomization/

15 exp randomized controlled trial/

16 exp "randomized controlled trial (topic)"/

17 exp placebo/

18 14 or 15 or 16 or 17

19 13 or 18

20 5 and 12 and 19

21 limit 20 to embase

**Search strategy of the Cochrane library**

EBM Reviews - Cochrane Central Register of Controlled Trials

EBM Reviews - Cochrane Database of Systematic Reviews

EBM Reviews - Cochrane Clinical Answers

EBM Reviews - Cochrane Methodology Register

EBM Reviews - Database of Abstracts of Reviews of Effects

1 (Vitamin D or Cholecalciferol or Cholecalciferols or Calciol or Vitamin D3 or Vitamin D 3).af.

2 (vitamin D or colecalciferol).sh.

3 1 or 2

4 (Reproductive Technique* or Reproduction Technique* or Reproduction Technic* or Reproductive Technology or Reproductive Technologies or Assisted Reproductive Technique* or Assisted Reproductive Technic* or Assisted Reproductive Technology or Assisted Reproductive Technologies or In Vitro Fertilization* or Test-Tube Fertilization* or Test Tube Fertilization* or Fertilizations in Vitro or Test-Tube Babies or Test-Tube Baby or Test-Tube Babies or Intracytoplasmic Sperm Injection* or ICSI).af.

5 (reproductive procedure or infertility therapy or in vitro fertilization or intracytoplasmic sperm injection).sh.

6 4 or 5

7 (random allocation or randomization or shame treatment or placebo or random*).af.

8 (randomization or randomized controlled trial or randomized controlled trial as topic or placebo).sh.

9 7 or 8

10 3 and 6 and 9
